# Novel identifications of cerebral hemodynamics using BOLD fMRI in patients with sickle cell disease

**DOI:** 10.1162/IMAG.a.1

**Published:** 2025-05-16

**Authors:** Brianna Kish, Jinxia (Fiona) Yao, Andrew John Frels, Jessica Budde, Vidhya Vijayakrishnan Nair, Andrew Q. Pucka, Ziyue Liu, Andrew RW O’Brien, Yunjie Tong, Ying Wang

**Affiliations:** Weldon School of Biomedical Engineering, Purdue University, West Lafayette, IN, United States; Department of Anesthesia, Stark Neurosciences Research Institute, Indiana University School of Medicine, Indianapolis, IN, United States; Indiana Center for Musculoskeletal Health, Indiana University, Indianapolis, IN, United States; Department of Biostatistics, Indiana University School of Medicine, Indianapolis, IN, United States; Division of Hematology/Oncology, Indiana University School of Medicine, Indianapolis, IN, United States

**Keywords:** sickle cell disease, hemodynamics, fMRI, arteriovenous shunting

## Abstract

Sickle cell disease (SCD) is a genetic blood disorder characterized by the production of abnormal hemoglobin known as hemoglobin S, which leads to reduced oxygen-carrying capacity of the blood. This reduced blood oxygenation can trigger cerebrovascular remodeling, leading to a higher risk of cerebrovascular disease and cognitive impairment. Despite growing evidence of the importance of cerebrovascular health in managing SCD, the lack of specific diagnostic tools makes this area an underutilized target in clinical care. In this cross-sectional study, we aimed to investigate the hemodynamic mechanisms of SCD through functional magnetic resonance imaging (fMRI) and their relationship with hematological parameters. In this pioneering study, we utilized the patterns of systemic low-frequency oscillations within the blood oxygen level-dependent fMRI signal to discern oxygen levels in the brain and characterize distinct blood flow patterns in patients with SCD. We formulated a unique model that revealed two blood flow patterns in SCD patients: firstly, an abnormal rapid flow pattern through arterio-venous shunting, where highly oxygenated blood reaches the superior sagittal sinus prematurely, circumventing most capillaries; secondly, a normal flow pattern, wherein normally oxygenated blood reaches the superior sagittal sinus after traversing through the capillaries. Our findings indicate that both flow patterns coexist in SCD patients, but in those with more severe blood abnormalities, the rapid flow pattern predominates. This study marks the first instance of employing fMRI to investigate the rich hemodynamic information in SCD patients. The results hold significant potential for the development of non-invasive hemodynamic biomarkers to gauge cerebrovascular health in SCD.

## 1 Introduction

Sickle cell disease (SCD) is a genetic blood disorder caused by a mutation in the beta-globin gene, leading to the production of abnormal hemoglobin (Hb) known as hemoglobin S (HbS) (Arkuszewski et al., 2013;DeBaun et al., 2006). This abnormal hemoglobin easily forms long, rigid chains, causing the formation of sickled erythrocytes which are prone to destruction, resulting in a reduced oxygen-carrying capacity of the blood. Consequently, SCD patients often have low hematocrit (Hct) levels and decreased oxygen affinity in their blood. To maintain proper oxygen delivery to the brain at rest, the body compensates by increasing cerebral blood flow (CBF) (Jordan et al., 2021;Juttukonda et al., 2017;Kassim & DeBaun, 2013). This can result in cerebrovascular shunting, a phenomenon where the increased blood velocity can adversely affect the efficiency of oxygen transfer in the brain’s capillaries. Studies have indicated that cerebrovascular shunting is more prevalent in individuals with SCD compared to those without the condition (Guilliams et al., 2015;Juttukonda et al., 2019). Over time, the continuous state of reduced oxygenation (chronic hypoxia) and shunting can lead to cerebrovascular remodeling, which can contribute to the development of cerebrovascular disease (Halder & Milner, 2021;Manwani & Frenette, 2013).

Functional magnetic resonance imaging (fMRI) is a non-invasive imaging technique widely used in neuroscience to indirectly measure brain function through blood flow. Blood oxygen level-dependent (BOLD) signal is the main contrast of fMRI that has the advantage of a high signal-to-noise ratio, and additional perfusion parameters can be derived using the BOLD signal, compared to arterial spin labeling imaging (Heeger & Ress, 2002;Matthews, 2004). The BOLD signal in fMRI is sensitive to the change in concentration of deoxyhemoglobin in the blood, which is paramagnetic. An increase in regional CBF brings in highly oxygenated blood leading to a decrease in the deoxyhemoglobin concentration, which causes a rise in the BOLD signal (Heeger & Ress, 2002;Matthews, 2004). Fluctuations in neuronal activity and physiology, for example, respiratory and cardiac frequencies, can drive these regional changes in CBF, causing BOLD signal fluctuations.

Systemic low-frequency oscillations (sLFOs) are a subset of low-frequency oscillations (0.01–0.1 Hz) that are widely represented throughout the brain with high correlations and meaningful delay values among each other in resting-state BOLD-fMRI data (Tong et al., 2017). Previous studies have investigated the mechanisms of sLFO signals and demonstrated the feasibility of using sLFO signals as an intrinsic “bolus” to track blood flow in the brain, especially via large vessels (Tong et al., 2019;Yao et al., 2019). Specifically, sLFOs between the internal carotid arteries (ICA) and the superior sagittal sinus (SSS) were negatively correlated with the ICA leading by ~5 s. This time difference between the arteries and the vein is consistent with the whole-brain transit time measured from a prior ultrasound study (Hoffmann et al., 2000), strongly suggesting that sLFOs from fMRI signals can be used as an intrinsic bolus to track blood flow. It is worth noting that the negative correlation is likely due to the significant difference in oxygen saturation between arterial and venous blood (Tong et al., 2019;Weisskoff & Kiihne, 1992). According to this model, it predicts a sign reversal in the BOLD signal when the oxygen saturation falls below an 86% threshold (Oxygen saturation in ICA: 100%; Oxygen saturation in SSS: 65–70%) or approximately halfway between arterial and venous oxygen saturation. The same study also found that the sLFO from the SSS and the Global Mean (GMean; averaged fMRI signal across the whole brain) were positively correlated (Oxygen saturation in the brain: 70–80%) with the GMean-sLFO leading by 3 s.

Considering SCD as a hematological disorder, it is hypothesized that alterations in CBF attributable to SCD should be detectable via BOLD fMRI signals. This study is designed to elucidate the hemodynamic underpinnings of sLFOs in SCD patients and delineate their correlation with laboratory biomarkers. To our knowledge, this is the first study to use fMRI to examine and evaluate the complex cerebrovascular changes under SCD. Previous fMRI work has only been used to study functional connectivity in the brains of SCD patients (Case et al., 2017;Karafin et al., 2019;McNeely et al., 2021;Singavi et al., 2016;Zempsky et al., 2017). We aim to develop a new noninvasive biomarker based on sLFOs that can assess the severity of cerebrovascular changes in SCD patients, to be verified through common hematological parameters. This will provide significant benefits by offering an early warning for cerebral vascular disease, stroke, and other related conditions. This could have tremendous benefits in helping determine the optimal use of disease-modifying therapies, particularly high-risk and intensive therapies like stem cell transplant and gene therapy.

## 2 Methods

### 2.1 Study participants and eligibility criteria

Data from 23 participants with SCD were included in the analyses for this cross-sectional study. Eligible participants were required to have a diagnosis of SCD and be between the ages of 14 and 80 years. Other eligibility criteria for the study included: 1) no significant visual, motor, or auditory impairment that would interfere with the ability to complete computer-based cognitive tests; 2) no recent (30 days) initiation or dose adjustment of stimulant medication; 3) no contraindications to MRI scans, including metallic implants or pregnancy; and 4) no use of pro re nata over the counter pain medications on the day of the MRI scan, nor caffeine or PRN opioid medications 24 h prior to MRI scan. Ten age-, sex-, and ethnicity-matched participants without SCD were enrolled as controls (Table 1). The study was approved by the Institutional Review Board at Indiana University. All participants provided written informed consent before enrollment.

**Table 1.** Subject demographic data

	Sickle cell disease (N = 23)		Healthy controls (N = 10)	
	(Mean ± SD) or n	Range or %	(Mean ± SD) or n	Range or %
Age, years	34.22 ± 13.00	14 - 73	36.91 ± 16.98	17 - 62
Females	13	56.52%	5	50%
Height, cm	171.57 ± 10.07	150.4 - 190.5	172.38 ± 7.26	159.6 – 184.4
Weight, kg	72.82 ± 17.40	37.6 - 112.6	76.46 ± 15.76	53.1 - 109
Hb, g/dL	9.70 ± 1.87	6.8 - 13.6	13.59 ± 1.34	12.2 - 16.5
Silent cerebral or other infarct	1	4.35%	N/A	N/A
Chronic transfusion therapy	3	13.04%	N/A	N/A
Hydroxyurea treatment	14	60.87%	N/A	N/A
SCD genotype (HbSS/Sβ0)	18	78.26%	N/A	N/A
SCD genotype (HbSC)	3	13.04%	N/A	N/A
SCD genotype (HbSβ+)	2	8.70%	N/A	N/A

### 2.2 Blood and hemoglobin examination

Laboratory blood testing, including complete blood cell count, reticulocyte count, and hemoglobin electrophoresis, was done upon enrollment. The following parameters were measured: white blood cell count, red blood cell count, hemoglobin level, hematocrit percentage, mean corpuscular volume, mean corpuscular hemoglobin, mean corpuscular hemoglobin concentration, red blood cell distribution width, platelet count, mean platelet volume, reticulocyte count, absolute reticulocyte number, hemoglobin F percentage, and hemoglobin S percentage.

### 2.3 MRI acquisition

All T1-weighted images and fMRI images were acquired on 3T SIEMENS (Prisma) with a 64-channel phased-array coil. The T1-weighted images were obtained with the parameters: TR = 2300 ms, TE = 2.98 ms, flip angle = 9 degrees, FOV = 240 mm x 240 mm x 250 mm with 1 x 1 x 1 mm as the voxel size. The BOLD functional MRI (fMRI) images were acquired at resting state with the parameters: TR = 1050 ms, TE = 29 ms, flip angle = 62 degrees, FOV = 90 mm x 90 mm x 60 mm, and 2.4 x 2.4 x 2.4 mm as the voxel size.

### 2.4 Vessel identification and fMRI data preprocessing

**Fig. 1.**
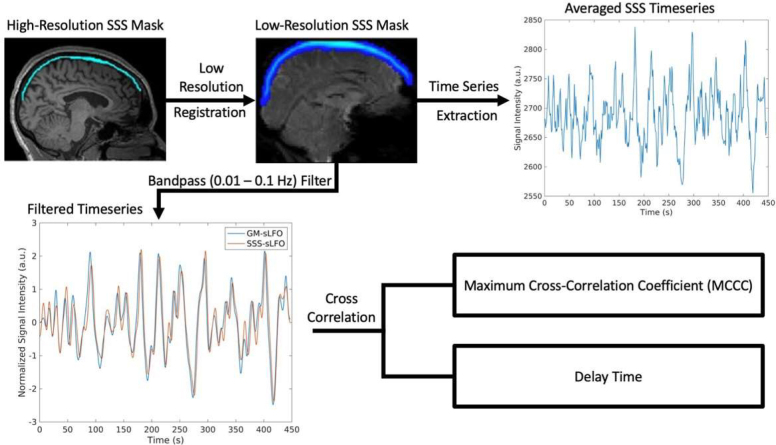
Flowchart of the MRI data processing pipeline. High-res T1w images are used to identify and mask the superior sagittal sinus (SSS). The mask is then transformed into the fMRI space, and the average signal is extracted. This signal and the global mean (GM) signal are bandpass filtered from 0.01–0.1 Hz to extract the systemic low-frequency oscillations (sLFO). The resulting waveforms are then cross-correlated to get the MCCC and delay time.

The fMRI data were preprocessed using the FMRIB Software Library (FSL) to correct for motion artifacts (Jenkinson et al., 2012). The first 10 volumes of the data were discarded to remove non-steady-state scans. Registration between the high-resolution images (T1w) and the low-resolution images (resting-state fMRI) was performed during preprocessing to obtain the transformation matrix. The superior sagittal sinus (SSS) was identified and manually masked from the T1w image using FSLeyes by a trained researcher, and blind verification was performed (Tong et al., 2019;Yao et al., 2019). The transformation matrix was then applied to the SSS mask from the T1w image to acquire the SSS mask in each subject’s fMRI space (Fig. 1).

### 2.5 Time series extraction and correlation (MCCC and CCC)

**Fig. 2.**
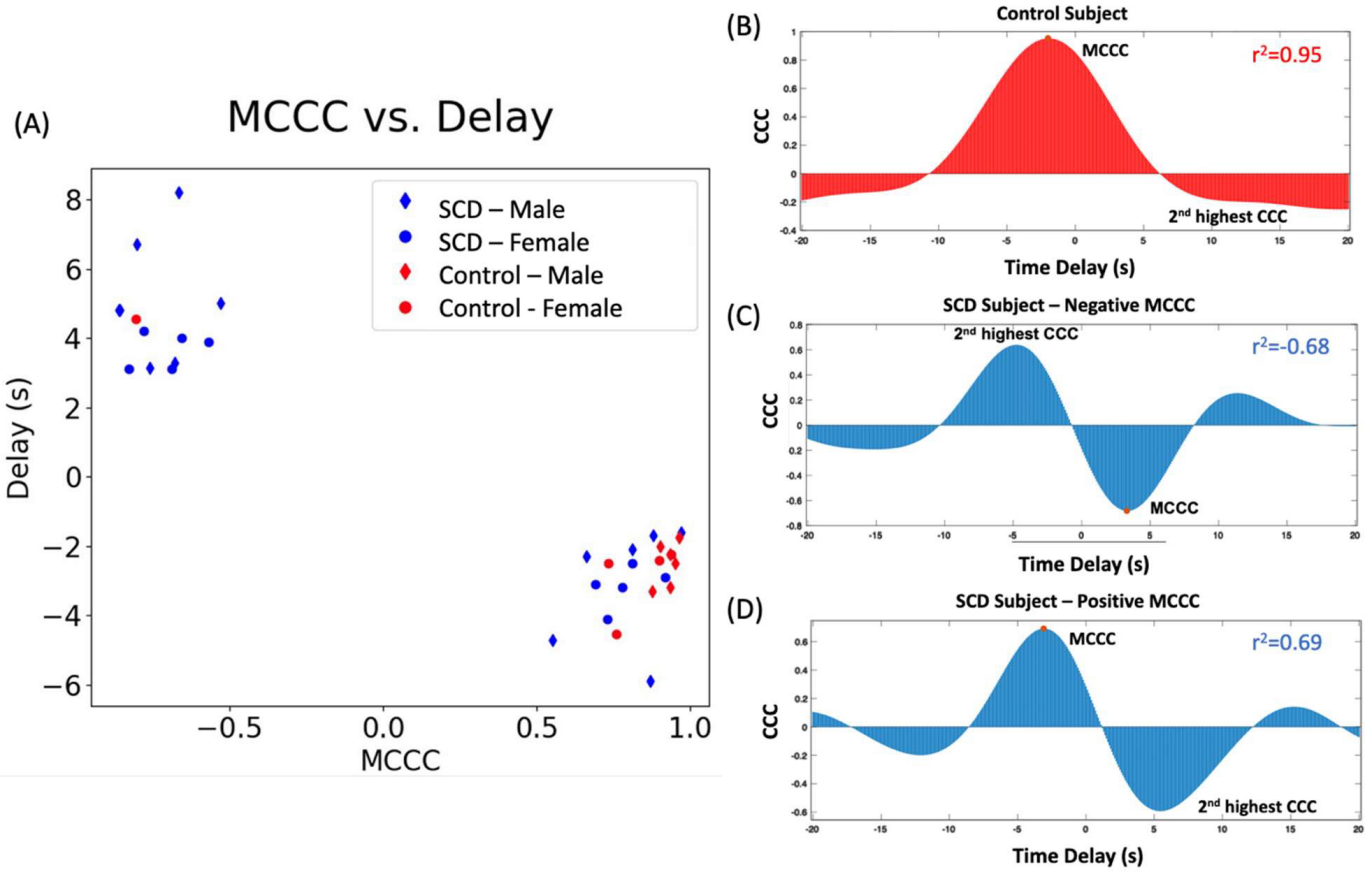
Determination of the maximum and 2^nd^highest cross-correlation coefficient (CCC) and corresponding delay times. (A) A scatter plot of the MCCC and the delay time values between the averaged SSS-sLFO and the GMean-sLFO for all SCD and unaffected subjects (males: ♦, females: •). All unaffected subjects (in red), excluding one, present high MCCC values with negative delay times. As for SCD (in blue), half of the patients behaved similarly to unaffected subjects with positive MCCC values and negative delay times, while half demonstrated the reverse. Example cross-correlation coefficient versus delay time plots for (B) unaffected, (C) negative MCCC SCD subject, and (D) positive MCCC SCD subject. MCCC highlighted by a red dot.

The time series extraction for the SSS and correlation procedure are the same as in previous publications (Tong et al., 2017,^2019^;Yao et al., 2019). In brief, the SSS mask was applied to the fMRI data to extract the mean SSS time series signal by the FSL function “fslmeants –w”. The global mean signal was generated by averaging the fMRI signal across the whole brain. Then, both signals were demeaned and underwent a bandpass filter (0.01–0.1 Hz) using MATLAB to acquire the sLFOs. Finally, a cross-correlation was performed between the sLFOs for the SSS and GMean with a constricted time window from -20 s to +20 s to calculate the maximum cross-correlation coefficients (MCCC) and their corresponding delay times (Fig. 2A).

The MCCC of the cross-correlation function identifies the precise moment when the signals are most closely aligned. This means that the temporal delay between the two signals can be determined by the position of this peak within the function. However, focusing solely on the single peak may neglect other significant temporal relationships between the two signals. To comprehensively evaluate these temporal dynamics, we also analyzed the entire CCC function across all subjects, with the findings presented inFigure 2B-D.

### 2.6 Statistical analysis

Pearson correlation was performed between the MCCC/delay times and the 14 different blood measurements, such as percentage of hemoglobin S, total amount of hemoglobin, and so on (full list shown inSupplementary Fig. 2), controlling for age and sex, and significant parameters (|r| > 0.3 and p < 0.05) were identified. With N = 23 SCD subjects, we have 80% power to detect the Pearson correlation coefficient with absolute values ≥0.55 at type I error 0.05.

Further hypothesis-generating analyses were performed based on whether MCCCs fell into the positive or negative groups. Unpaired t-tests assuming equal variance were used to determine significant differences in these parameters between subjects with negative or positive MCCCs. With N = 11 and N = 12 subgroups of SCD subjects (MCCC+, MCCC- groups respectively), we have an 80% power to detect effect sizes ≥1.23 using Student’s t-tests. Linear regression was performed between significantly correlated hematological indexes and MCCC and delay times in the positive and negative SCD MCCC groups and healthy controls separately (Fig. 4;Supplementary Fig. 3).”

## 3 Results

### 3.1 Negative MCCCs found in half of SCD subjects

InFigure 2A, a scatter plot is presented, showing the relationship between MCCC values and their corresponding delay times between the averaged SSS-sLFO and the GMean-sLFO, computed for each individual.

#### 3.1.1 Expected

The majority of unaffected subjects, except for one individual (seeSupplementary Fig. 1), exhibited high positive MCCC values with negative delay times (Table 2), indicating that the GMean-sLFO led the averaged SSS-sLFO. In contrast, 11 out of 23 SCD patients exhibited similar MCCCs and delay times to unaffected subjects (Table 2). The observed MCCC and delay, with the SSS lagging behind the brain, indicates the predominance of a regular blood flow path from the brain to the SSS, with comparable oxygen saturation levels (Tong et al., 2019;Weisskoff & Kiihne, 1992).

**Table 2.** Average cross-correlation coefficient and corresponding delays for the MCCC and 2^nd^highest peak in sample sub-groups

	SCD: positive MCCC	SCD: negative MCCC	Unaffected controls
MCCC	0.889 ± 0.080	-0.748 ± 0.105	0.735 ± 0.516
MCCC Delay (s)	-2.67 ± 0.816	3.708 ± 0.710	-2.014 ± 2.310
2 ^nd^ highest CCC	-0.533 ± 0.186	0.463 ± 0.144	-0.275 ± 0.285
CCC Delay (s)	7.75 ± 2.476	-8.214 ± 4.372	9.045 ± 7.281

#### 3.1.2 Unexpected

The 12 remaining SCD patients showed a negative MCCC value between the averaged SSS-sLFO and the GMean-sLFO, accompanied by positive delay times (Table 2), suggesting that the SSS-sLFO led. This outcome is unforeseen. The observed delay, marked by the SSS preceding the GMean, implies that blood reaches the SSS prior to traversing the brain.

### 3.2 Second CCC peak found in SCD subjects, with reversed signs

Figure 2shows the CCC function for a typical unaffected control (Fig. 2B) and two representative SCD patients with positive and negative MCCCs inFigure 2C-D.

#### 3.2.1 Expected

In the unaffected control group, the CCC function typically yields a single, unequivocal maximum point (Table 2) stemming from one prominent peak within the temporal window of ±20 s. This singular peak distinctly marks a clear and definitive time delay between the two signals. The characteristic of having a singular, dominant peak in the CCC function is the predominant finding in unaffected controls (Table 2). This observation aligns with the patterns noted in our prior studies involving healthy subjects (Tong et al., 2019).

#### 3.2.2 Unexpected

InFigure 2C-D, the CCC spectra of two representative SCD subjects, characterized by positive and negative MCCC values, display two distinct peaks. In subjects with a negative MCCC, the second-highest peak corresponds to a substantial positive CCC (|r| > 0.3;Yao et al., 2019) and a negative delay (Table 2), whereas in subjects with a positive MCCC, it corresponds to a substantial negative CCC and a positive delay. A negative CCC implies differing oxygen saturation between the brain and SSS, with blood reaching the SSS before perfusing the brain. In contrast, a positive CCC suggests similar oxygen saturation levels and that blood reaches the brain before the SSS. This dual-peak pattern may reflect two temporally distinct blood arrival pathways to the SSS.

For SCD subjects with a positive MCCC (Fig. 2D), the second-highest peak reveals a substantial (|r| > 0.3), negative CCC, coupled with a positive delay. A single control subject exhibited a similar pattern (Supplementary Fig. 1). The negative CCC and positive delay indicate the oxygen saturation levels in the brain and SSS are different, and that oxygenated blood reaches the SSS, bypassing the brain tissue.

Remarkably, the pattern of dual peaks, characterized by a reversed sign in CCC and a corresponding delay in the second peak, was consistently observed across all SCD subjects, encompassing both the positive and negative MCCC groups, as detailed inTable 2. The conventional calculation of MCCC might overlook this distinctive feature, which was not evident in the averaged data from unaffected controls (avg |r| < 0.3), as indicated inFigure 2BandTable 2.

### 3.3 Correlations between blood measurements and fMRI metrics

The correlation matrix of all 14 hematological indexes to the derived MCCC and delay values is shown inSupplementary Figure 2. Of particular interest are the variables Hb and HbS, due to their high and significant correlation (|r| > 0.3 and p < 0.05) to the MCCCs and delays and their known relation to SCD.

**Fig. 3.**
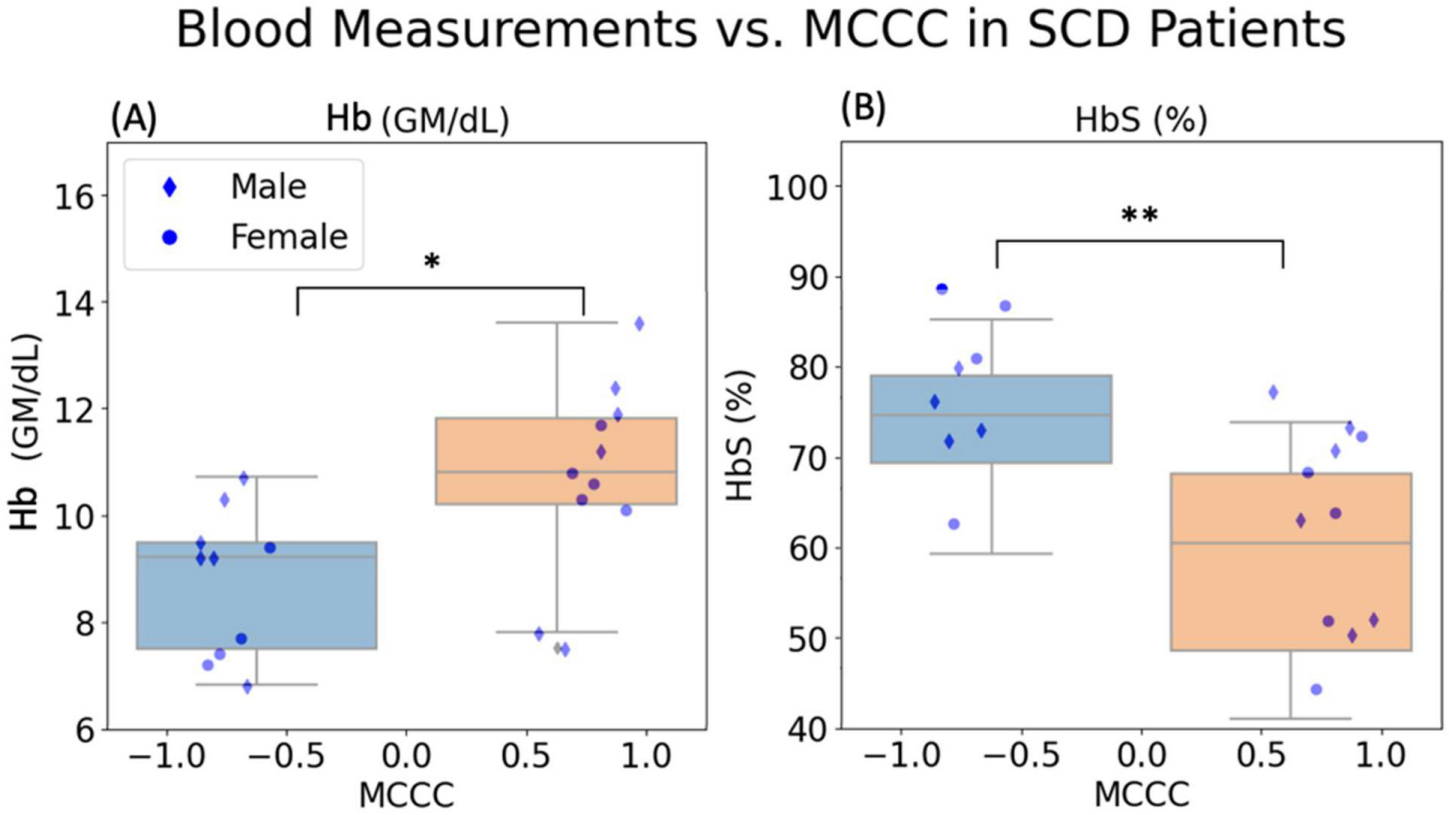
Comparison of significantly correlated blood measurements to MCCC values. Blood measurements (A) Hb and (B) HbS (%) and MCCC values for all SCD subjects. SCD patients with negative MCCC values are shown to have significantly lower Hb and Hct values and significantly higher HbS values than those with positive MCCCs (Female: circles; Male: diamonds).

The relationships between the two key hematological indexes and MCCCs in SCD patients are illustrated inFigure 3. Patients with negative MCCCs have significantly lower Hb (p = 0.011) (Fig. 3A) and higher HbS (p = 0.004) (Fig. 3B) as compared to those with positive MCCCs. Interestingly, all subjects with negative MCCCs have either HbSS or HbSβ0 type SCD (10 SS and 2 Sβ0).

**Fig. 4.**
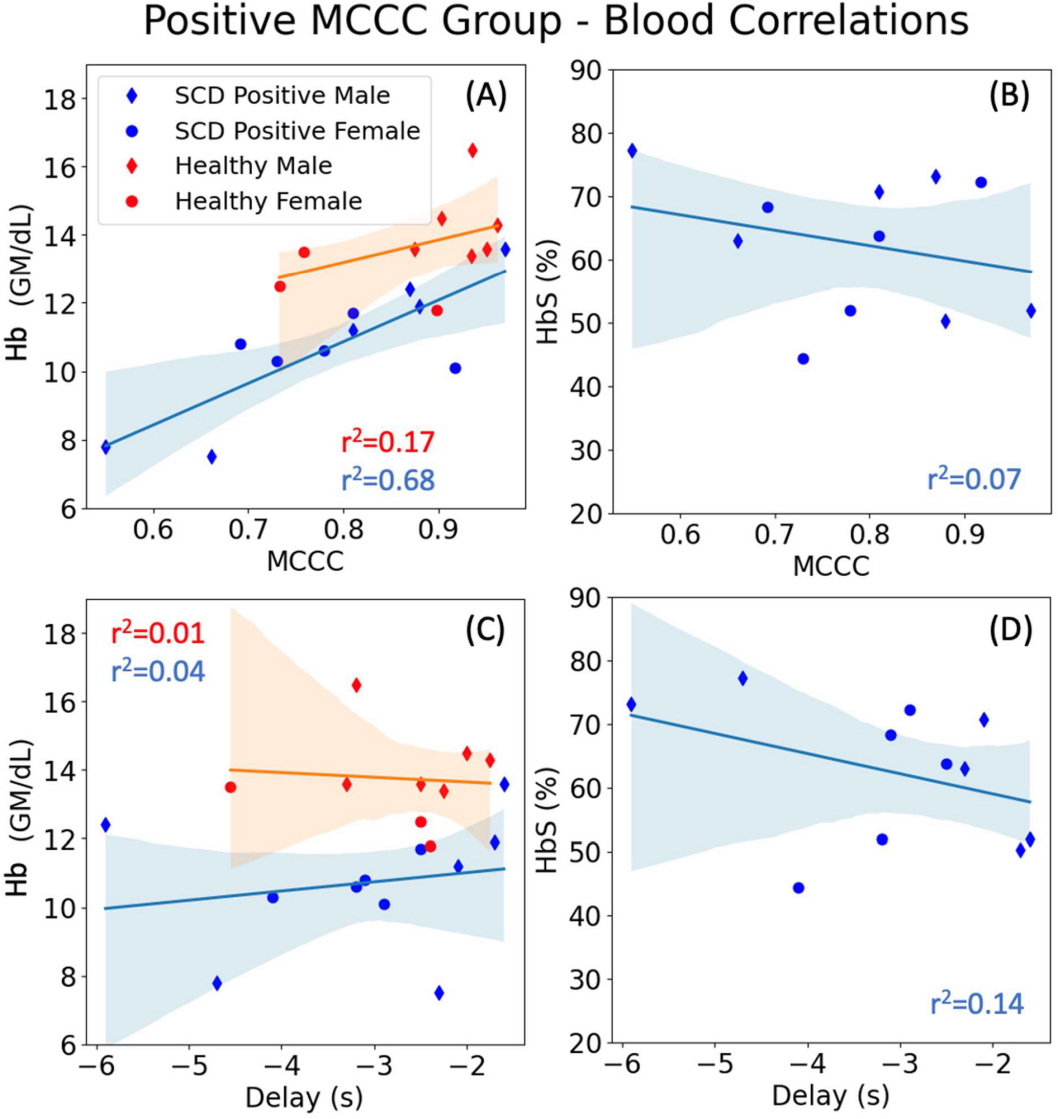
Linear correlation between blood measurements and fMRI metrics in the positive MCCC groups. Correlations between blood measurements (A, C) Hb and (B, D) HbS and MCCC and delay values for unaffected (red) and SCD (blue) subjects. 95% confidence intervals shown in corresponding color-shaded regions. Only SCD subjects with positive MCCC values are shown. The percentage of Hb exhibit high correlation with MCCC values in SCD with a p-value less than 0.05. HbS shows a moderate correlation with MCCC values and delay times as well.

Intriguing results were also observed in subjects (both unaffected and with SCD) exhibiting positive MCCCs (Fig. 4).Figure 4A-Bshows the correlation between the patients’ positive MCCC values and specific blood measurements, whileFigure 4C-Ddisplays the correlation between the patients’ delays and these blood measurements. A significant positive linear correlation was observed between Hb and MCCC values in positive MCCC SCD patients (r^2^= 0.676, p < 0.05). However, unaffected subjects showed a non-significant linear correlation (r^2^= 0.1729, p > 0.05). These findings suggest that, for individuals with positive MCCCs, higher Hb values are associated with higher MCCC values, and this trend is only significant in SCD patients. Additionally, HbS exhibited a loose negative correlation to MCCC values in positive MCCC SCD patients (r^2^= 0.0729, p > 0.05). However, among subjects with negative MCCCs, Hb and HbS were not linearly correlated with MCCC and delays (Supplementary Fig. 3).

The correlations between the delays and Hb in the same cohort (i.e., unaffected and SCD patients with positive MCCCs) are significantly weaker (Unaffected: r^2^= 0.0078, p > 0.05, SCD: r^2^= 0.038, p > 0.05). Additionally, Hb values of the unaffected subjects (indicated by red dots inFig. 4) are within normal ranges and are not significantly correlated with delay values (GMean-sLFO leading: -2 to -4 s). In contrast, SCD patients exhibit lower Hb values (as expected), which show a weak positive correlation with delays, indicating that higher Hb values are associated with shorter delays. The range of delay values is also wider (i.e., -2 to -6 s). However, the correlation between HbS and delays is not significant, similar to the case with MCCCs (r^2^= 0.144, p = 0.2632).

## 4 Discussion

### 4.1 Novel fMRI-BOLD signal model

#### 4.1.1 Background of the model

In this study, we introduce a novel model aimed at elucidating variations in the fMRI-BOLD signal observed in SCD patients. Before we introduce the model, it is worth emphasizing the premises of this fMRI study: Firstly, the sLFO BOLD is recognized as an intrinsic natural bolus, extractable from various brain structures, and capable of monitoring blood flow. Secondly, the global mean of sLFOs (GMean) denotes the mean oscillatory activity across the brain, whereas sLFO from SSS signifies oscillations within the SSS. By conducting a cross-correlation analysis of these two measurements, we can deduce the temporal delay, which reflects the sequence and duration of blood flow. Thirdly, the magnitude of the MCCC can be either positive or negative, indicating differences in oxygenation which would arise from preferential fast blood flow (negative MCCC) or normal blood flow (positive MCCC) (Tong et al., 2019;Weisskoff & Kiihne, 1992). It is of note that the BOLD signals used in this analysis are derived from susceptibility differences between oxygenated and deoxygenated hemoglobin; however, SCD subjects possess altered hemoglobin, HbS. While preliminary evidence suggests that there is no susceptibility difference between the two, potential differences could alter BOLD signal amplitude (Eldeniz et al., 2021). However, our analyses focus on the relation between two signals rather than absolute signal amplitude. In this scenario, we might experience lowered sensitivity, but our findings remain interpretable.

#### 4.1.2 Introduction of the new model

**Fig. 5.**
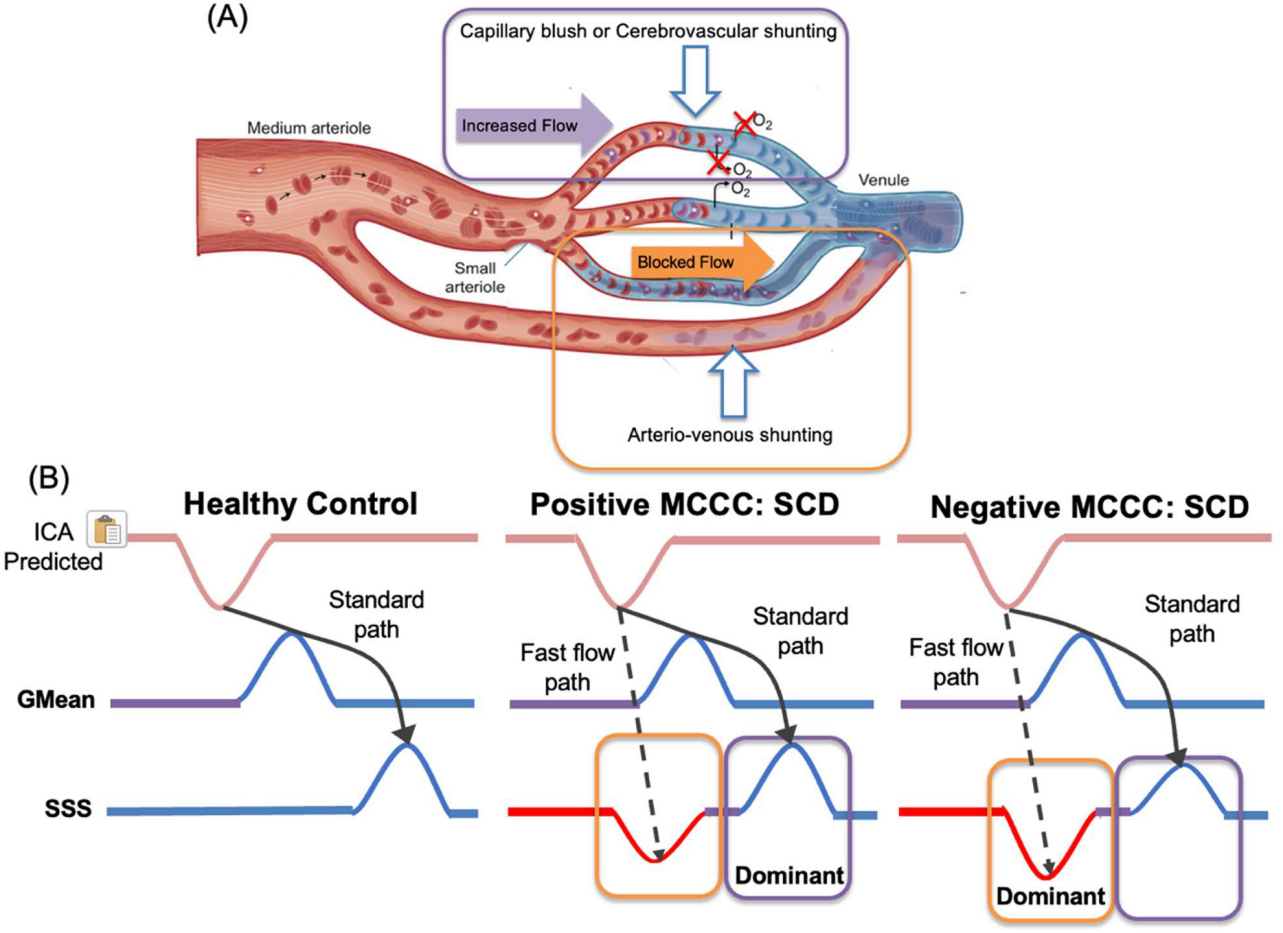
fMRI model for oxygen saturation in BOLD signal and possible physiological shunting mechanisms. (A) Diagram of different shunting mechanisms. Capillary blush scenario circled in purple. Increased cerebral blood flow leads to increased capillary velocity, reducing oxygen offloading capacity. Arteriovenous shunting scenario circled in orange. Blocked capillary flow leads to increased resistance and a switch to faster microvascular shunt flow (Figure repurposed with edits from (Detterich et al., 2019)). (B) Three shunting scenarios are depicted. In the healthy subject, little to no shunting is expected, thus blood flow follows one path from the internal carotid artery (ICA) to the global mean (GMean) to superior sagittal sinus (SSS), and the GMean and SSS are positively correlated. In the positive MCCC sickle cell subject, shunting leads to two blood flow paths. The first travels from ICA, skips the majority of capillary flow, and ends in the SSS. The blood is highly oxygenated (red) and thus the GMean and SSS are negatively correlated. The second path travels from the ICA through the capillary bed, and results in deoxygenated (blue) blood in the SSS, thus the GMean and SSS are positively correlated. In this scenario, the second path is dominant, thus the MCCC is positive. In the third scenario, the two blood flow paths exist as well; however, flow in the first path is dominant, thus the MCCC is negative.

This model specifically accounts for the unique variations in blood oxygenation and flow that occur in patients with SCD that has never been reported in previous studies. InFigure 5A, we demonstrate possible cerebral blood flow changes under SCD. The fundamental and most important part of the model is that we introduce a dual hemodynamic pathway for SCD patients, in which we argue that oxygen-rich blood from the arteries, such as ICAs, takes two distinct pathways to the venules and veins (Fig. 5):

1. *Normal Flow Pathway*: Oxygen-enriched arterial blood circulates through the brain, sequentially flowing through arterioles, capillary beds, and venules, before ultimately draining into larger veins such as the SSS. According to the model, arterial blood with high oxygen levels (over 90%) flows into the brain. As this blood distributes oxygen, its oxygen saturation decreases, resulting in a decline in GMean oxygenation (reversing the signal from that of ICA). This same blood continues its journey into the veins but with a delay and further reduced oxygen saturation. Consequently, the MCCC between the signals in GMean and SSS is positive but exhibits a negative delay, indicating that GMean is leading.
2. *Abnormal “Shunted” Flow Pathway*: Here, the arterial blood either circumvents the capillaries, progressing directly to the venules and veins and/or rushes through a localized region of capillaries in a process called shunting. This divergence leads to the arrival of highly oxygenated blood at the SSS much sooner than usual. It is important to note that this blood does not traverse through the majority of the capillary bed and hence does not majorly contribute to the GMean, which is dominated by capillary signals. Instead, the GMean primarily reflects the blood following the standard flow pathway (as mentioned earlier). Consequently, when comparing the GMean with this accelerated signal in the SSS, the MCCC demonstrates a negative value attributable to the increase in fractional shunt volume. Additionally, the positive delay indicates that this highly oxygenated blood reaches the SSS considerably earlier than the blood taking the regular pathway.

It is postulated that SCD patients engage both pathways, with a fraction of the oxygen-laden arterial blood adhering to the conventional route while the rest opt for the expedited shunt pathway. As indicated, the fMRI signal in the SSS comprises both signals, arriving asynchronously and exhibiting opposite signs. The allocation of blood across these pathways is not consistent but fluctuates according to variables like total Hb, and HbS concentrations, among others. The congruence between our model’s predictions and empirical fMRI data substantiates the model’s validity.

#### 4.1.3 Physiological evidence for the model

The enduring observation regarding hemodynamics in SCD we hypothesize involves the phenomenon of early venous filling (EVF). Early venous filling refers to the early appearance of blood in the cerebral venous pathways before the conclusion of the arterial phase, without the presence of large arteriovenous malformations or fistulas. This pattern of EVF in the SSS and vein of Galen is noted frequently in patients following thrombectomies of the major cranial arteries and is postulated to occur due to some combination of two predominant theories (Dorn et al., 2012;Elands et al., 2021;Ohta et al., 2004;Shimonaga et al., 2020). The first hypothesizes that following the removal of the thrombosis, there occurs a regional “capillary blush” wherein the blocked blood flow rushes through the regional vascular pathways at an increased rate leading to early arrival at the major veins (Fig. 5A). The second theorized method is that blood flows through arteriovenous (A-V) shunts. These are small, non-gas exchanging, direct connections from the arteries to veins that bypass the capillary bed (Fig. 5A) (Detterich et al., 2019;Fritzsch et al., 2015;Nemoto et al., 2013;Nemoto & Bragin, 2022;Rowbotham & Little, 1965;Zhang et al., 2018). Both theories offer two distinct “pathways” for blood to flow: one of slow flow through the capillary bed and one resulting from fast regional flow and/or A-V shunt flow, which could lead to two “arrival peaks,” in the major draining veins.

In the context of SCD patients, they suffer from increased CBF and acute vaso-occlusive events, in which sickled erythrocytes block significant capillary flow (Fig. 5A). In severe cases, blockages can happen in the major arteries (Darbari et al., 2020;Guilliams et al., 2017;Veluswamy et al., 2019). Due to the transient nature of these events, SCD patients are under a repetitive cycle of partially blocked, then released, blood flow with a higher base CBF. Though differing from the more atherosclerotic nature of previous cases of EVF, we hypothesize that this cycle produces similar effects to that of patients recently recovering from thrombectomies, inducing or encouraging these capillary blush and A-V shunting phenomena. Specifically, we would like to highlight A-V shunting, since increased capillary resistance encourages the switch from standard flow to microvascular A-V shunt flow (Nemoto et al., 2013;Nemoto & Bragin, 2022). Transiently or chronically blocked vessels and increased CBF, both seen in SCD, would increase this capillary resistance. Examples of A-V shunting in other vascular beds (e.g., lungs, heart, limbs) have been thoroughly documented in SCD; however, the presence of shunting in the central nervous system for SCD is still poorly understood (Dowling et al., 2010;Hambley et al., 2019;Nahavandi et al., 2002;Veluswamy et al., 2019).

These results and hypotheses align closely with work from Jesperson and Østergaard, in which they propose that the cerebral metabolic rate of oxygen is dependent on both CBF and cerebral transit time heterogeneity (CTTH) (Jespersen & Østergaard, 2012). Specifically, CTTH refers to varied blood transit times through the capillary bed. This phenomenon has been verified in the mouse cortex with optical imaging (Merkle & Srinivasan, 2016). Jesperson and Østergaard demonstrate through modeling that high CTTH leads to less oxygen deposition within the tissues. Increased shunting in SCD would increase the CTTH, agreeing with our hypothesis that shunting decreases oxygen deposition.

In the remainder of this discussion, we will interpret the results through the lens of this model, and toward the end, we will address its limitations and its impact on SCD imaging studies.

### 4.2 Correlation and anti-correlation explained by dual-pathway hemodynamic model

#### 4.2.1 Dual CCC peaks in SCD subjects

Examination ofFigure 2andTable 2reveals a distinctive characteristic of dual peaks in the CCC function, consistently present in SCD patients but absent in all but one control subject. These peaks are notably similar in their significant values. Crucially, each pair comprises a positive peak accompanied by negative delays and a negative peak associated with positive delays. 1) The presence of a positive peak with negative delays suggests that a particular sLFO reaches the SSS later than it does in the brain (GMean), carrying oxygen saturation levels similar to or lower than those in the brain. This pattern signifies that the blood is following the regular flow route, moving from the ICA to the brain and then to the SSS. 2) Conversely, a negative peak accompanied by positive delays signifies that a specific sLFO reaches the SSS earlier than it does in the brain (GMean), and it exhibits higher oxygen saturation levels than in the brain. This indicates that blood preferentially takes the fast pathway described in the model. It is important to note that while the dual-peak pattern is a consistent finding in SCD subjects, one control participant also exhibited this feature (Supplementary Fig. 1). As discussed previously, the fast-flow pathway may result from various physiological conditions affecting cerebrovascular health that are not specific to SCD—such as anemia. However, the presence of abnormal blood pathology in SCD appears to consistently produce this phenomenon. Our research study presents a promising fMRI biomarker that holds the potential to facilitate the study of hematological diseases.

#### 4.2.2 Correlate with blood measures

The presence of dual peaks in the CCC function, observed in SCD patients and near-absent in controls, suggests that rapid blood flow is a prevalent characteristic in the SCD cohort but not in the control group. Typically, the MCCC value is influenced by the predominant peak within the CCC function, signifying the dominance of a particular flow mechanism. InFigure 2A, we note that 12 SCD individuals exhibit negative MCCC values alongside positive delays, denoting a predominance of the rapid flow path in these cases. Conversely, the remaining 11 SCD subjects present with positive MCCC values and negative delays, indicating that in these instances, regular blood flow path is the predominant mechanism.Figure 3showcases the blood metrics in SCD patients and delineates their association with the primary flow mechanism. Notably, SCD patients exhibiting dominance of the fast flow pathway display markedly reduced levels of Hb and HCT, alongside elevated HbS concentrations. Notably, the 12 subjects exhibiting negative MCCCs have HbSS or HbSβ0 type SCD, typically considered the most “severe” form of SCD (Quinn, 2016;Sridhar & Idowu, 2021). Congruently, the presence of intracardiac or pulmonary shunts has been observed in high percentages in sample groups with HbSS-type SCD (Dowling et al., 2010;Hambley et al., 2019;Nahavandi et al., 2002).

SCD patients present with atypical blood profiles, characterized by diminished Hb, reduced HCT, and the presence of HbS, in contrast to healthy controls. The study further reveals a direct correlation: as the deviation in blood matrices from the norm intensifies in SCD patients, the fraction of faster flow correspondingly escalates, which might have ties to SCD type.

#### 4.2.3 MCCCs linearly correlated with hematological parameters

Interestingly, the 12 negatively correlated SCD patients exhibited lower Hb/Hct and higher HbS than the SCD patients who had positive correlations. Even among SCD patients with positive MCCCs (Fig. 4A-C), the measures of Hb/Hct are significantly positively linearly correlated with MCCCs, while the measure of HbS is weakly negatively correlated with MCCCs. This might indicate that the degree of reduced Hb/prevalence of HbS is associated with the split of flow between the two mechanisms (Konotey-Ahulu, 2005;Platt et al., 1994;Scott & Scott, 1999). This indicates the possibility that, as blood flow splits between the two pathways, the synchronization of sLFOs between voxels across the brain is likely to diminish, reducing overall GMean coherence (i.e., out of phase LFOs will cancel each other when averaging) and causing two distinct blood arrival times at the SSS. This would reduce the observed MCCC value. Additionally, as shown inFigure 4, a comparable, but non-significant linear relationship is observed between positive MCCCs and Hb/Hct in unaffected controls, supporting that this dual flow pathway only appears more frequently in SCD.

### 4.3 Limitations and future work

Several limitations need to be addressed in this study. Firstly, it is a recognized phenomenon that in SCD patients, cerebral blood flow (CBF) escalates to compensate for their diminished oxygen-carrying capacity. Similar to “capillary blush,” it has been proposed that global CBF can increase to the point at which there is decreased oxygen offloading efficiency in the capillary bed, leading to higher oxygenation in the venous system throughout the brain, termed cerebrovascular shunting (Juttukonda et al., 2017,^2019^,^2021^). Under this model, we anticipated that even the standard flow would exhibit accelerated flow rates, potentially resulting in reduced time delays between the brain and the SSS in both CCC peaks. Nevertheless, our observations, as depicted inFigure 2andTable 2, reveal that these time delays closely align with those of the control subjects, a finding that remains not fully understood. One hypothesis is that the convergence of sLFO at the SSS, arriving via diverse routes and timings, might lead to a “smoothing” effect on the aggregated SSS signals, thereby extending the delay duration for the first and second peaks. To counteract this, we could introduce modulations to the sLFO through methods like breath-holding, aimed at reducing the periodicity of sLFO and enhancing the precision of the CCC and delay measurements.

Furthermore, our SCD cohort included individuals undergoing different treatment methods for SCD, hydroxyurea (n = 12), chronic blood transfusions (n = 1), and hydroxyurea and chronic blood transfusions (n = 2). These treatments directly affect the patient’s blood makeup and, therefore, should affect the results of our cross-correlation analysis. To address these effects, we performed logistic regression on all 23 subjects to compare MCCC and delay to treatment methods. No significant relationships were identified. Additionally, we performed the same analysis within the MCCC+ and MCCC- groups separately. Subjects with hydroxyurea treatment exhibited shorter delay times, but only within the MCCC+ group (p = 0.0314), indicating that the treatment may increase blood flow speed but only along the normal flow pathway. However, with the low number of subjects, further research is needed to draw conclusive evidence.

sLFOs exhibiting high oxygenation levels are initially expected to manifest in the ICAs (Yao et al., 2019). As per the model, these sLFOs subsequently propagate through two distinct pathways. Incorporating data on sLFOs from the ICAs is a critical step toward further substantiating the model. In the current study, the Field of View (FOV) was insufficiently expansive to encompass the necessary segments of the ICAs. Future studies employing larger imaging FOVs that include the ICAs would facilitate a more comprehensive validation of our hypotheses. Lastly, the sample size of the patient population is relatively small, and further research should be performed to address any susceptibility differences between Hb and HbS. Despite these limitations, this study represents the first investigation into sLFO dynamics in SCD and uses fMRI signals to explain physiological connections. While further validation and the formulation of new hypotheses are required, these findings have important implications for the application of fMRI in SCD research, opening new avenues for investigation in this field.

## 5 Conclusions

In conclusion, the present study employed fMRI to investigate the hemodynamics of SCD patients and identified several unexpected results. We propose a dual-pathway blood flow model that suggests the presence of variable degrees of shunting in SCD. In our model, more severe anemia, that is, reduced Hb and HCT and increased HbS, is associated with higher degrees of shunting. While further validation and refinement of the model are required, this suggests that BOLD-fMRI can be leveraged for assessing vascular pathways in SCD patients, a diagnostic approach unprecedented in this field. These findings offer valuable insight into the complex pathophysiology of SCD and show promise as a potential novel biomarker of SCD pathology.

## Supplementary Material

Supplementary Figure 1

Supplementary Figure 2

Supplementary Figure 3

## Data Availability

All data and codes are available by reasonable request to Y.W.
