# Supplementary material for: Novel identifications of cerebral hemodynamics using BOLD fMRI in patients with sickle cell disease
Source: Imaging Neurosci (Camb). 2025 May 16;3:IMAG.a.1. doi: 10.1162/IMAG.a.1 (PMC12319992; doi:10.1162/IMAG.a.1)
Supplement: Supplementary Figure 1 [file imag.a.1_suppfig1.pdf]

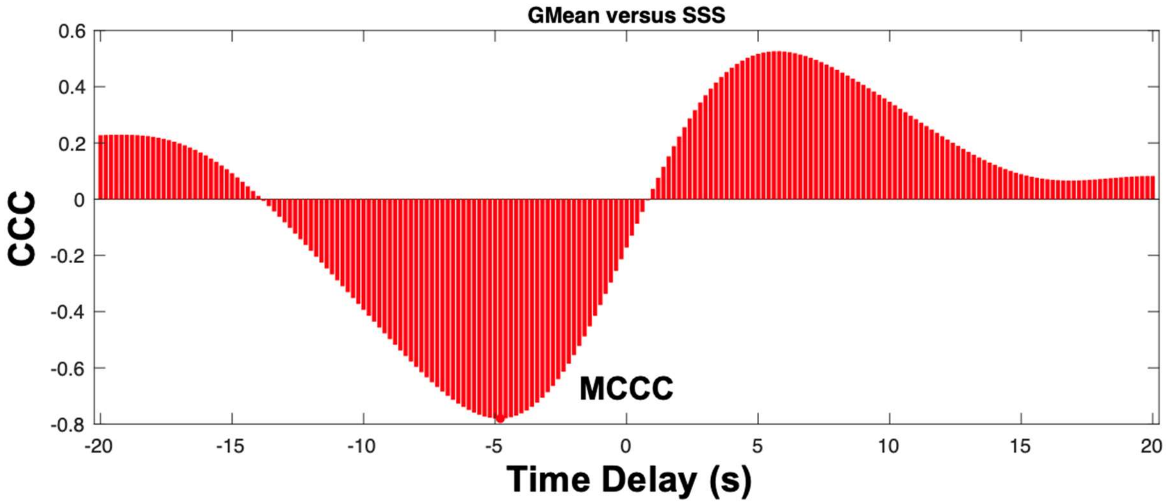

Supplementary Figure 1. Example cross-correlation coefficient vs. delay time plot for the only unaffected subject with a negative MCCC (red dot) and negative delay value. The plot shows the dual-peak pattern seen in SCD subjects with significant peaks in both negative and positive CCC values (CCC>0.3).
