# Supplementary material for: Novel identifications of cerebral hemodynamics using BOLD fMRI in patients with sickle cell disease
Source: Imaging Neurosci (Camb). 2025 May 16;3:IMAG.a.1. doi: 10.1162/IMAG.a.1 (PMC12319992; doi:10.1162/IMAG.a.1)
Supplement: Supplementary Figure 2 [file imag.a.1_suppfig2.pdf]

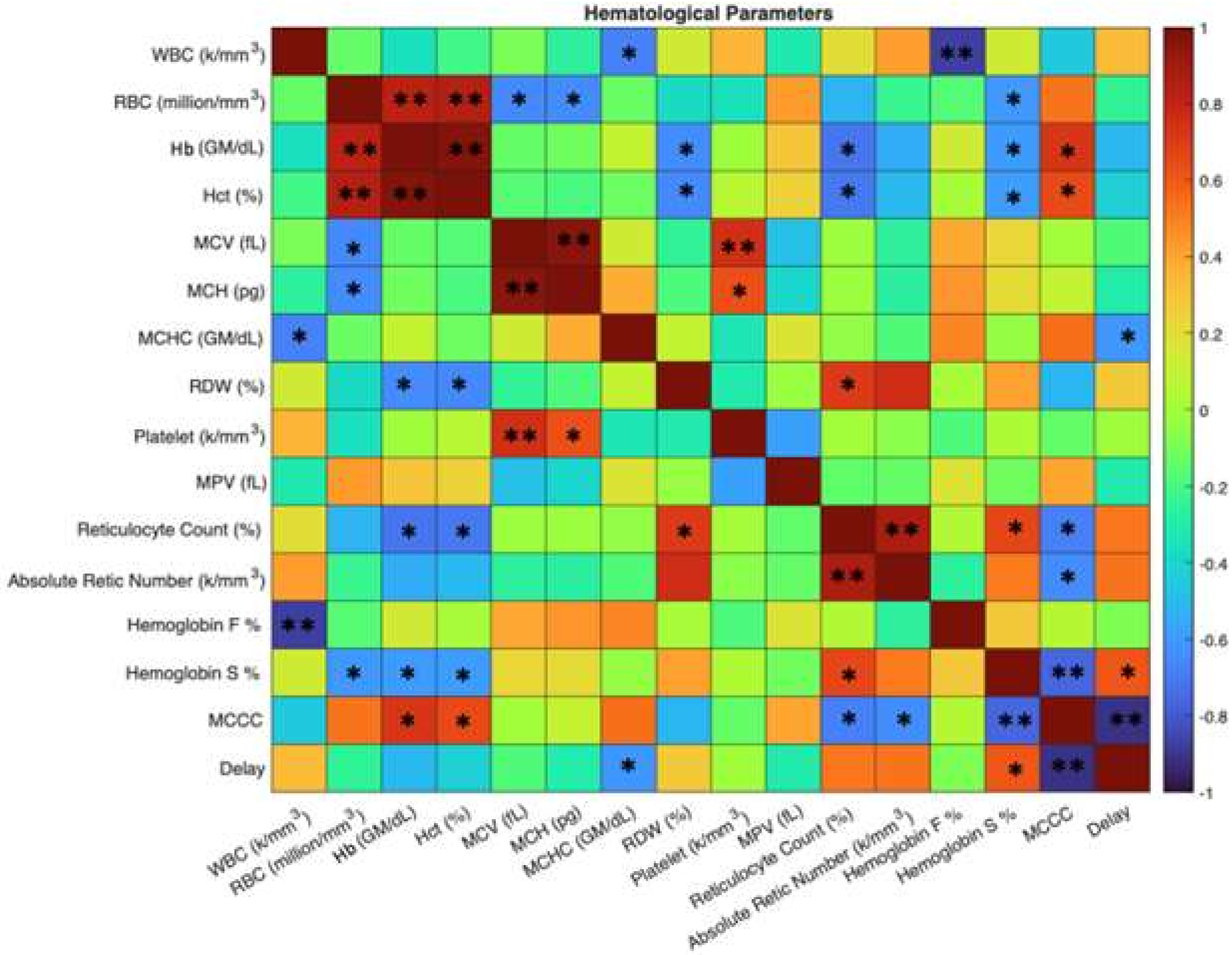

Supplementary Figure 2. Correlation matrix of all 14 blood measurements and additional variables to derived MCCC and delay values (color represents r, * for $p<0.05$, ** for $p<0.01$). The 14 measured blood metrics were WBC (k/cumm), RBC (million/cumm), Hb (GM/dL), Hct (%), MCV (fL), MCH (pg), MCHC (GM/dL), RDW (%), Platelet (k/cumm), MPV (fL), Reticulocyte count (%), Absolute Retic number (k/cumm), Hemoglobin F (%), Hemoglobin S (%). Age (yrs) and Sex were controlled for as covariates. As shown, high correlation values ($r>0.3$) were found between MCCC/delay and WBC, Hb, Hct, MCHC, Reticulocyte count, Absolute Retic Number, and Hemoglobin S, and significant correlation values ($p<0.05$) were found between MCCC/delay and Hb and Hct.
