# Supplementary material for: Novel identifications of cerebral hemodynamics using BOLD fMRI in patients with sickle cell disease
Source: Imaging Neurosci (Camb). 2025 May 16;3:IMAG.a.1. doi: 10.1162/IMAG.a.1 (PMC12319992; doi:10.1162/IMAG.a.1)
Supplement: Supplementary Figure 3 [file imag.a.1_suppfig3.pdf]

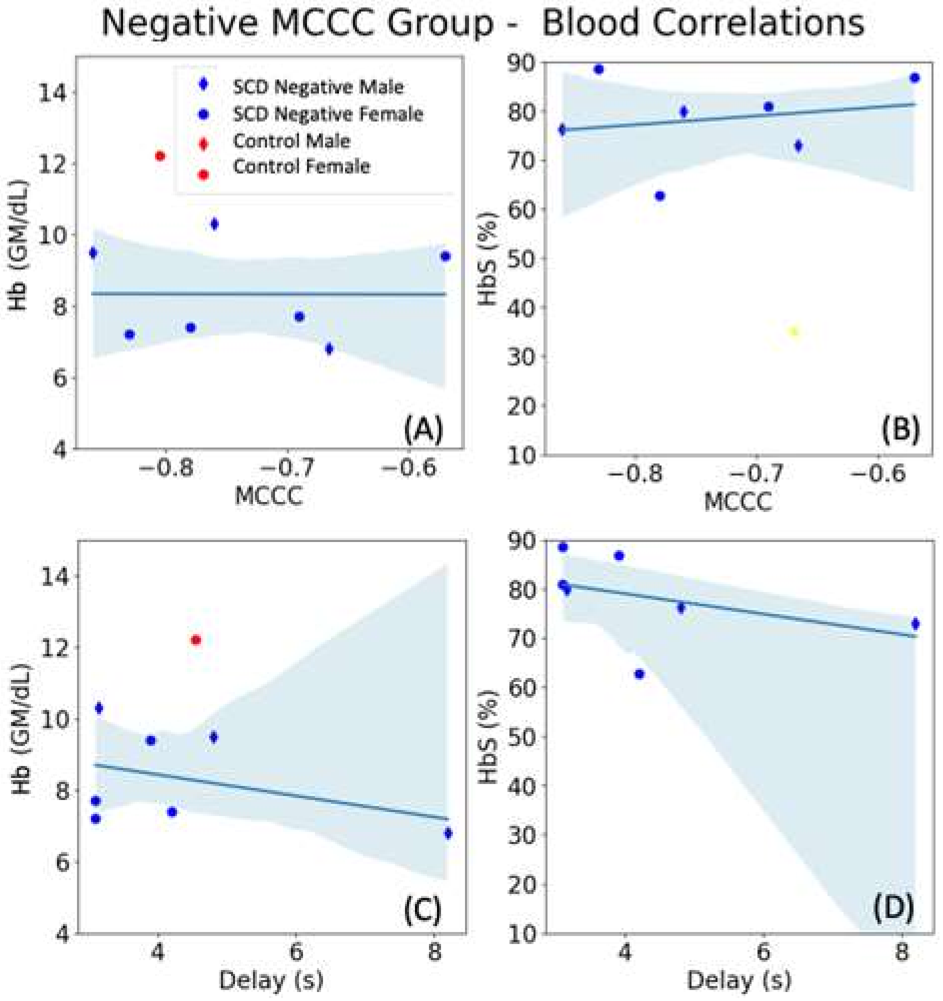

Supplementary Figure 3. Correlations between blood measurements (A, C) Hb and (B, D) HbS and MCCC and delay values for unaffected (red) and SCD (blue) subjects. 95% confidence intervals are shown in corresponding color-shaded regions. Only subjects with negative MCCC values are shown. The percentage of HbS and total Hb exhibit a moderate correlation with MCCC values and a weak correlation with delay times.
